# Human defensins 5 and 6 enhance HIV-1 infectivity through promoting HIV attachment

**DOI:** 10.1186/1742-4690-8-45

**Published:** 2011-06-14

**Authors:** Aprille Rapista, Jian Ding, Bernadette Benito, Yung-Tai Lo, Matthew B Neiditch, Wuyuan Lu, Theresa L Chang

**Affiliations:** 1 Public Health Research Institute, University of Medicine and Dentistry of New Jersey-New Jersey Medical School, Newark, NJ 07103, USA; 2 Department of Microbiology and Molecular Genetics, University of Medicine and Dentistry of New Jersey-New Jersey Medical School, Newark, NJ 07103, USA; 3 Institute of Human Virology and Department of Biochemistry and Molecular Biology, University of Maryland School of Medicine, Baltimore, MD 21201, USA; 4 Department of Epidemiology and Population Health, Albert Einstein College of Medicine, Bronx, NY 10461, USA

## Abstract

**Background:**

Concurrent sexually transmitted infections (STIs) increase the likelihood of HIV transmission. The levels of defensins are frequently elevated in genital fluids from individuals with STIs. We have previously shown that human defensins 5 and 6 (HD5 and HD6) promote HIV entry and contribute to *Neisseria gonorrhoeae*-mediated enhancement of HIV infectivity *in vitro*. In this study, we dissect the molecular mechanism of the HIV enhancing effect of defensins.

**Results:**

HD5 and HD6 primarily acted on the virion to promote HIV infection. Both HD5 and HD6 antagonized the anti-HIV activities of inhibitors of HIV entry (TAK 779) and fusion (T-20) when the inhibitors were present only during viral attachment; however, when these inhibitors were added back during viral infection they overrode the HIV enhancing effect of defensins. HD5 and HD6 enhanced HIV infectivity by promoting HIV attachment to target cells. Studies using fluorescent HIV containing Vpr-GFP indicated that these defensins enhanced HIV attachment by concentrating virus particles on the target cells. HD5 and HD6 blocked anti-HIV activities of soluble glycosaminoglycans including heparin, chondroitin sulfate, and dextran sulfate. However, heparin, at a high concentration, diminished the HIV enhancing effect of HD5, but not HD6. Additionally, the degree of the HIV enhancing effect of HD5, but not HD6, was increased in heparinase-treated cells. These results suggest that HD5 and haparin/heparan sulfate compete for binding to HIV.

**Conclusions:**

HD5 and HD6 increased HIV infectivity by concentrating virus on the target cells. These defensins may have a negative effect on the efficacy of microbicides, especially in the setting of STIs.

## Background

There were an estimated 33 million people living with HIV in 2007, and there were 2.7 million new HIV infections, with the predominant mode of infection being sexual transmission (UNAIDS 2008). Currently, there is no effective vaccine or microbicide available to prevent HIV spread. According to CDC data in 2008, approximately 56,000 people become newly infected with HIV every year in the U.S. It was estimated that more than 21% of the 1.1 million infected individuals in the U.S. are unaware of their infection. While the spread of HIV is inefficient, sexually transmitted infections (STIs) are known to increase the likelihood of HIV transmission [1-5].

Defensins are antimicrobial peptides important to innate mucosal immunity [6-9]. Indeed, the levels of defensins in genital fluid are frequently elevated in individuals with STIs [10-13], suggesting a potential role of defensins in modulating HIV transmission. Recently, antimicrobial peptides including human neutrophil defensins 1-3 (HNPs 1-3) and LL-37 have been found to be increased in cervicovaginal secretions from women with STIs and are independently associated with increased HIV acquisition [14]. While HNPs 1-3 and LL-37 exhibit anti-HIV activities *in vitro *(reviewed in [15,16]), other human alpha-defensins such as human defensins 5 and 6 (HD5 and HD6), enhance HIV infectivity *in vitro *[17]. Increased levels of HD5 have been reported in urethral secretions of men with *Neisseria gonorrhoeae *and *Chlamydia trachomatis *infection [12] and in cervicovaginal secretions from women with bacterial vaginosis (BV) [18], indicating a possible role of defensins in enhanced HIV transmission by STIs and BV.

HD5 and HD6 are constitutively expressed by intestinal Paneth cells and play an important role in gut mucosal immunity [6-9]. HD5 is also found in cervical lavage fluid as well as in the female genital tract [18,19], and gene expression of HD5 and HD6 can be detected in cervicovaginal epithelial cell lines [17]. Concentrations of HD5 protein ranging from 1 to 50 μg/ml have been reported in diluted vaginal fluid from healthy women [18,19]. We have recently shown that HD5 and HD6 significantly enhance HIV infection at the step of viral entry [17]. Enhancement of HIV infection was observed with primary HIV isolates in primary CD4+ T cells. Induction of HD5 and HD6 in response to gonococcal infection increased HIV infectivity, suggesting a role of defensins in STI-mediated increased HIV transmission [17]. Importantly, our recent in vitro study has shown that HD5 and HD6 can antagonize anti-HIV activity of polyanionic microbicides including PRO2000, cellulose sulfate, and carrageenan [20]. These polyanionic microbicides failed to protect women against HIV infection in several clinical trials [21-23]. Although the contributions to the ineffectiveness of these microbicides are likely multifactorial, mucosal host factors such as HD5 and HD6 may have a potential negative effect on the efficacy of microbicides.

Here, we dissected the molecular mechanisms by which HD5 and HD6 enhance HIV infectivity. Our results demonstrated that HD5 and HD6 promoted HIV attachment. Both HD5 and HD6 negated anti-HIV activities of soluble glycosaminoglycans (GAGs), although HD5, but not HD6, may compete with heparin/heparan sulfate for binding to HIV. The consequence of elevated levels of defensins in response to STIs may lead not only to increased susceptibility to HIV infection, but also to ineffectiveness of polyanion-based microbicides.

## Results

### Pre-incubation of HIV with defensins significantly increased HIV infection

**Figure 1:**
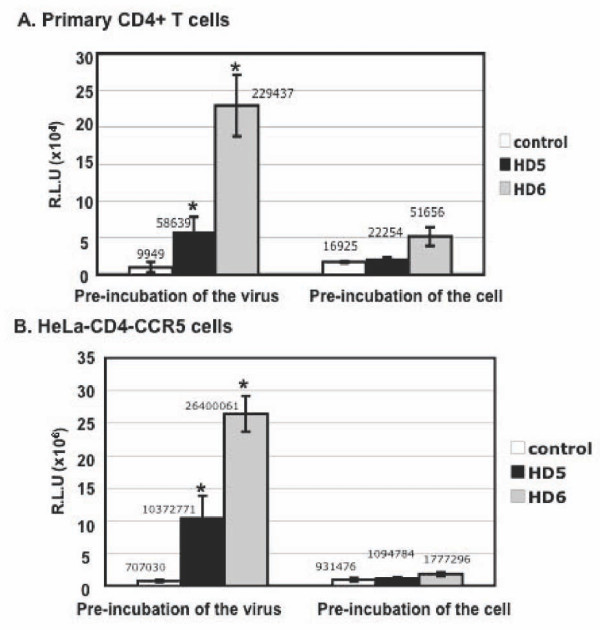
**HD5 and HD6 enhanced HIV infectivity by targeting the virus**. Pseudotyped HIV-1_JR-FL _luciferase reporter virus was incubated with or without HD5 or HD6 (20 μg/ml) at 37°C for 1 hour followed by infection of primary CD4+ T cells (A) or HeLa-CD4-CCR5 cells (B) as described in Materials and Methods. To determine the effect of defensins on the target cell, primary CD4+ T cells or HeLa-CD4-CCR5 cells were incubated with defensins in the presence of FBS for 1 hour, washed, and exposed to pseudotyped HIV-1_JR-FL _reporter virus for 2 hours. Cells were washed and cultured for 48 hours before measuring luciferase activity. Difference between defensin-treated virions and non-treated control was significant (*p < 0.05) as calculated by two-tailed, paired Student ***t ***test. The value of mean luciferase readout is shown. Data are means ± SD of triplicate samples and represent three independent experiments.

We have previously shown that HD5 and HD6 increase HIV infection when HIV is pre-treated with defensins [17]. Additionally, defensins do not affect HIV infection after cells are exposed to the virus, suggesting that these peptides act on HIV entry. To dissect the mechanism of this HIV enhancing effect, we first examined whether defensins enhanced HIV infection by acting on the virion or the target cell. Pseudotyped HIV-1_JR-FL _luciferase reporter virus was incubated with HD5 or HD6 for 1 hour before addition to PHA-activated primary CD4+ T cells (Figure 1A) or HeLa-CD4-CCR5 cells (Figure 1B). After 2 hours of incubation, infected cells were washed and cultured for 48 hours before measurement of luciferase activity. To assess the effect of defensins on the target cell, activated primary CD4+ T cells (Figure 1A) or HeLa-CD4-CCR5 cells (Figure 1B) were treated with defensins for 1 hour followed by washing extensively before exposure to pseudotyped HIV-1_JR-FL _luciferase reporter virus for 2 hours. Luciferase activity was determined 48 hours after infection. HIV infection was significantly increased by 6 to 15-fold with HD5 and by 23 to 37-fold with HD6 in both primary CD4+ T cells and HeLa-CD4-CCR5 cells when the HIV virion was pre-incubated with defensins. Note that the degree of HIV enhancing effect of defensins (20 μg/ml, equivalent to 5.6 μM for HD5 and 5.4 μM for HD6) varied from 6 to 40-fold, possibly due to the different virus stocks and the target cell condition (e.g. cell passage). Nevertheless, the results of enhancement of HIV infection by HD5 and HD6 were consistent. HD5 did not increase HIV infection when cells were pre-treated with defensins. HD6 slightly promoted HIV infection of activated CD4+ T cells (by ~3-fold), but had no effect on HIV infection of HeLa-CD4-CCR5 cells. The degree of enhancement of HIV infectivity by defensins was significantly higher when the HIV virion was pre-incubated with defensin compared to pre-incubation of cells. We conclude that HD5 and HD6 primarily acted on the virion to achieve their HIV enhancing effect.

### HD5 and HD6 negated the activity of HIV entry and fusion inhibitors

**Figure 2:**
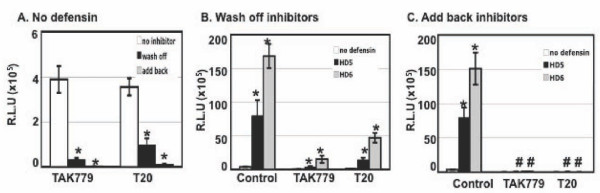
**HD5 and HD6 negated the activity of HIV entry and fusion inhibitors**. HeLa-CD4-CCR5 cells were pre-treated with or without TAK-779 (2 μM) or T-20 (200 nM) for 1 hour. Pseudotyped HIV-1_JR-FL _virus was incubated with HD5 or HD6 at 20 μg/ml at 37°C for 1 hour. The virus mixture was then added to HeLa-CD4-CCR5 cells in the presence or absence of inhibitors for 2 hours. After washing off unbound virus, infected cells were cultured in the (B) absence (wash off) or (C) presence (add back) of the inhibitors (TAK-779 or T-20) for 48 hours before measurement of luciferase activity. Differences between HIV inhibitor-treated samples vs no inhibitor control in panel A were significant (*p < 0.05). Difference between samples with and without treatment of defensins in panel B was also significant (*p < 0.05). When HIV inhibitors were added back to the cells after viral attachment at 37°C, the difference between samples with and without defensin treatment was not significant (#*p *> 0.05). Data are means ± SD of triplicate samples and represent three independent experiments.

Because HD5 and HD6 promote HIV entry, we addressed whether these defensins interfered with anti-HIV activities of inhibitors for HIV entry and fusion. HeLa-CD4-CCR5 cells were pretreated with TAK779, which is a small molecule targeting HIV co-receptor CCR5, or were pretreated with T-20, which blocks HIV fusion. Cells without treatment with HIV inhibitors were also prepared as a control. Pseudotyped HIV-1_JR-FL _luciferase reporter virus was incubated with or without defensins for 1 hour. The virus mixture was then added to the pretreated target cells and incubated for 2 hours. Cells were washed and cultured for 48 hours either in the absence (Figure 2B) or presence of added back HIV inhibitor (Figure 2C). As expected, TAK779 and T20 inhibited HIV infection, and the inhibitory effect was more potent (more than 99%) when the inhibitors were added back after viral attachment (Figure 2A). When HIV inhibitors were present only at the step of viral attachment, HD5 and HD6 abolished anti-HIV activities of TAK779 and T20 (Figure 2B). However, TAK779 and T20 overrode the HIV enhancing effect of defensins when the inhibitors were added back after viral attachment (Figure 2C). These results indicated that mucosal innate effectors such as HD5 and HD6 could negatively impact the efficacy of entry and fusion inhibitors under certain conditions.

### HD5 and HD6 increased HIV attachment to target cells

**Figure 3:**
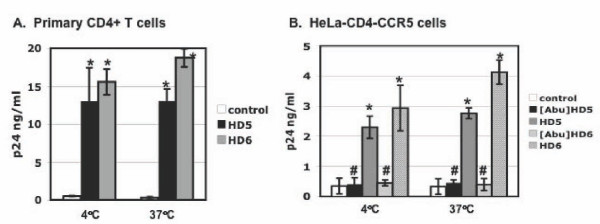
**HD5 and HD6 enhance HIV attachment to target cells**. Pseudotyped HIV-1_JR-FL _virus was incubated with HD5 or HD6 at 20 μg/ml as well as their linear analogs, [Abu]HD5 and [Abu]HD6, at 37°C for 1 hour, added to (A) PHA-activated primary CD4+ T cells (5 × 10^5 ^per sample) or (B) HeLa-CD4-CCR5 cells (5 × 10^4 ^per sample). Cells were incubated with defensins at 4°C or 37°C for 1 hour, washed extensively with PBS and lysed with Triton X-100. The level of cell-associated HIV p24 was determined by ELISA. Difference between defensin-treated virions and non-treated control was significant (*p < 0.05), whereas the difference between samples with and without treatment with linear peptides [Abu]HD5 and [Abu]HD6 was not significant (#*p *> 0.05). Data are means ± SD of triplicate samples and represent three independent experiments.

To delineate specific steps of the HIV life cycle influenced by defensins, we investigated the effect of HD5 and HD6 on HIV attachment at 4°C and 37°C. Incubation at 37°C leads to HIV internalization by target cells. Pseudotyped HIV-1 _JR-FL _luciferase reporter virus was incubated in the presence or absence of defensins for 1 hour. As a comparison, we also included identically charged linear, unstructured analogs of HD5 and HD6, [Abu]HD5 and [Abu]HD6 [24]. We have previously shown that [Abu]HD5 and [Abu]HD6 do not exert any HIV enhancing effect [17]. The virus-defensin mixture was added to HeLa-CD4-CCR5 cells at 4°C or at 37°C for 1 hour. Unbound virus was washed extensively before measurement of cell-associated HIV p24 by ELISA. HD5 and HD6 enhanced HIV attachment at 4°C or at 37°C to both activated CD4+ T cells (Figure 3A) and HeLa-CD4-CCR5 cells (Figure 3B). The linear analogs [Abu]HD5 and [Abu]HD6 did not exhibit any effect on HIV attachment to target cells (Figure 3B), indicating that the enhancing effect of defensins on HIV attachment required a properly folded structure of defensins.

**Figure 4:**
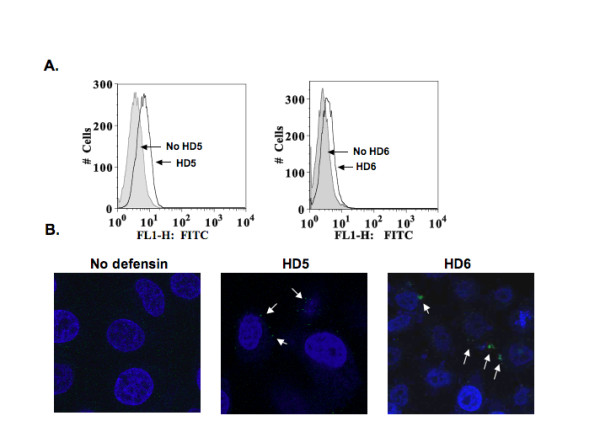
**HD5 and HD6 promote attachment of fluorescent Vpr-GFP-labeled virions to the target cells**. Pseudotyped HIV-1_JR-FL _virus containing Vpr-GFP was incubated with or without HD5 and HD6 at 20 μg/ml at 37°C for 1 hour before addition to HeLa-CD4-CCR5 cells. After 2 hours incubation at 4°C, cells were extensively washed with cold-PBS, fixed, and analyzed by FACS (A) or microscopy (B). In panel A, the gray histogram represents the signal from samples without defensins, whereas the open histogram represents the signal from cells with exposure to defensin-treated fluorescent HIV. In panel B (magnification, 40X), white arrows indicate concentrated fluorescent HIV.

To further confirm the enhancement of HIV attachment by defensins, fluorescent HIV virions containing Vpr fused with green fluorescent protein (GFP) were treated with or without HD5 or HD6 followed by incubation with target cells at 4°C. HIV attachment was assessed by FACS analysis or confocal microscopy. Although a previous report by Zhang *et al*. [25] demonstrated the attachment of fluorescent virions to CHO cells in the absence of serum using deconvolution microscopy, in our experiment there was no detectable signal in cells with exposure to HIV-1_JR-FL _Vpr-GFP virus in the presence of FBS, determined by FACS analysis or confocal microscopy. Interestingly, the fluorescent signal was significantly increased on cells with exposure to defensin-treated virus (Figure 4A). Similarly, the attachment of HIV-1_JR-FL _Vpr-GFP virus to cells was not apparent when the fluorescent virions were not treated with defensins (Figure 4B left panel). However, fluorescent dots were evident on cells with exposure to defensin-treated virions (Figure 4B), suggesting that defensins concentrated the virions on the target cell.

### The role of glycosaminoglycans (GAGs) in defensin-mediated enhancement of HIV infection

**Figure 5:**
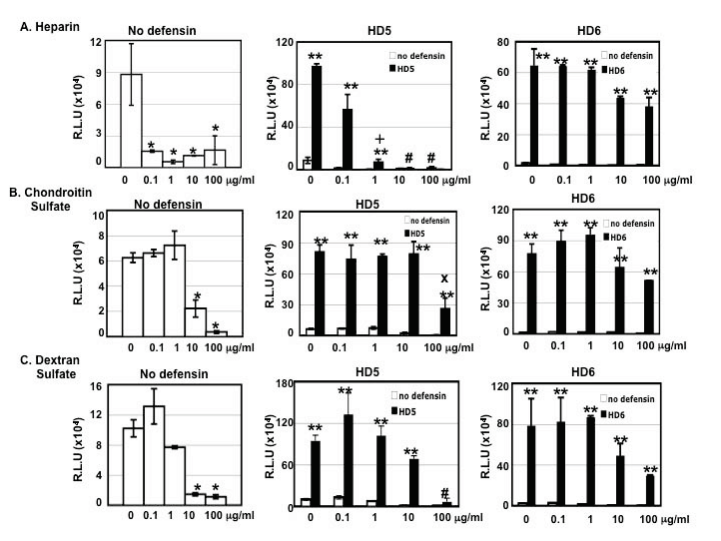
**Effect of soluble GAGs on defensin-mediated enhancement of HIV infectivity**. Pseudotyped HIV_-JR-FL _virus was incubated with or without HD5 or HD6 at 20 μg/ml in the absence or presence of heparin (A), chondroitin sulfate (B), and dextran sulfate (C) at various concentrations. After washing off unbound virus, infected cells were cultured for 48 hours before measurement of luciferase activity. Anti-HIV activities of soluble GAGs in the absence of defensins are shown in the left panel. Black bars represent the effect of soluble GAGs on HIV enhancement by HD5 (middle panels) and HD6 (right panels). Open bars (in the middle panel) represent samples in the absence of defensins. In the left panels, the difference between soluble GAG-treated virions and non-treated control is significant (*p < 0.05). In the middle and right panels, the difference between samples with or without defensins is significant (**p < 0.05) except samples treated with heparin at 10 or 100 μg/ml or dextran sulfate at 100 μg/ml in the presence of HD5 (#*p *> 0.05). After Bonferroni correction, the difference between heparin (1 μg/ml)-treated samples with or without HD5 was not significant (+, *p *= 0.06). Similarly, the difference between condroitin sulfate (100 μg/ml)-treated samples with or without HD5 was not significant (x, *p *= 0.14) after Bonferroni correction. Data are means ± SD of triplicate samples and represent three independent experiments.

GAGs such as heparan sulfate and chondroitin sulfate, which are widely expressed on the cell surface, are important for HIV attachment and infection [25-27]. We investigated the role of soluble GAGs including heparin, chondroitin sulfate, and dextran sulfate in defensin-mediated enhancement of HIV infection. In agreement with previous reports [28-32], heparin, chondroitin sulfate, and dextran sulfate exhibited anti-HIV activities (Figure 5A-C, left panels). HD5 at 20 μg/ml abolished anti-HIV activity of heparin at 0.1 μg/ml (equivalent to 6 nM, based on the molecular weight of 16 kD), but not at higher concentrations (10 and 100 μg/ml) (Figure 5A. middle panel). In contrast, HD6 at 20 μg/ml abolished anti-HIV activities of heparin at all tested concentrations of heparin (Figure 5A, right panel). Both HD5 and HD6 blocked anti-HIV activity of chondroitin sulfate, although chondroitin sulfate at 100 μg/ml reduced the HIV enhancing effect of HD5 and HD6 (Figure 5B). Similarly, HD5 and HD6 abolished anti-HIV activity of dextran sulfate (Figure 5C), although dextran sulfate at 100 μg/ml completely attenuated the HIV enhancing of HD5 and reduced the effect of HD6 by 60%. These results indicate that GAGs more effectively attenuated the HIV enhancing effect of HD5 than of HD6.

**Figure 6:**
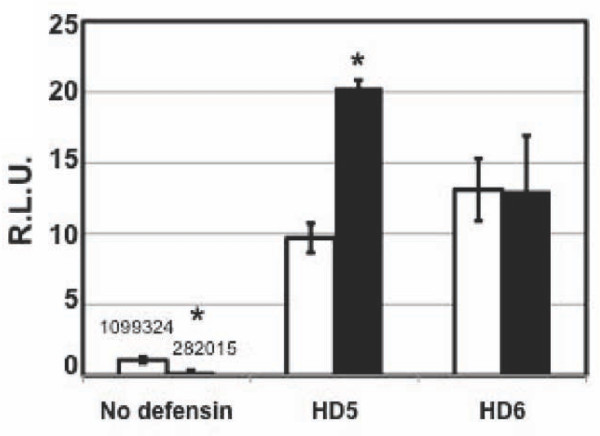
**Defensin-mediated enhancement of HIV infection in heparinase I-treated cells**. HeLa-CD4-CCR5 cells were treated with heparinase I at 37°C for 2 hours to remove cell-associated heparin and heparan sulfate (3:1). Cells were washed followed by exposure to defensin-treated pseudotyped HIV_-JR-FL _luciferase reporter virus for 2 hours. Infected cells were cultured for 48 hours. Difference between samples in cells with or without heparinase treatment was indicated (*, *p*<0.05). Data are means ± SD of triplicate samples and represent two independent experiments.

To determine the impact of cell-associated GAGs on the enhancement of HIV infection by defensins, HeLa-CD4-CCR5 cells were treated with heparinase I, which removes heparin and heparan sulfate and blocks HIV attachment [33]. Cells were washed with PBS and then exposed to HIV with or without defensin treatment. As expected, heparinase treatment significantly reduced HIV infection by 73-84% (Figure 6 and data not shown). The degree of enhancement of HIV infection by HD5 was further increased in heparinase-treated target cells by 2-fold compared to that in cells without heparinase treatment. In contrast, heparinase treatment did not affect the HIV enhancing effect of HD6. These results suggest that HD5 and heparin/heparan sulfate may compete for the same regions of HIV.

## Discussion

We demonstrated that HD5 and HD6 enhanced HIV infectivity by promoting virion attachment, a rate-limiting step of HIV entry [34]. These defensins appeared to increase HIV attachment by concentrating virions on the target cell. HD5 and HD6 negated the anti-HIV activity of HIV entry and fusion inhibitors, TAK 779 and T20 when the inhibitors were present only during viral attachment. While both defensins antagonized anti-HIV activities of several soluble GAGs, the HIV enhancing effect of HD5, but not HD6, was sensitive to heparin at higher concentrations. Additionally, the removal of cell-associated heparin/heparan sulfate led to an increase in enhancement of HIV infection by HD5, but not HD6, suggesting that these two defensins interact differently with HIV.

Alpha-defensins are structurally similar, despite their moderate sequence identity and distinct cellular functions [35]. For example, unlike all other alpha-defensins, HD6 exhibits little antibacterial activity [36]. HNPs1-4 inhibit HIV infection [15,37], whereas HD5 and HD6 promote HIV infection [17]. Although both HD5 and HD6 are Paneth cell defensins, their amino acid sequences have little homology beyond a few conserved residues: six Cys residues, an Arg-Glu salt bridge [38], and an invariant Gly residue [39]. These results suggest that specific residues in defensins may make subtle contributions to their structures resulting in distinct functions. Defensins may aggregate virions through oligomerization as illustrated by the recently reported self-association ability of HD5 [40], and HD6 may assemble into an elongated, high-order helical structure [35]. The structural findings are consistent with our observation that HD6 has a strong tendency to self-associate in solution and to form high-order aggregates on target molecules (personal communication to W. Lu). We speculate that higher-order HD6 aggregates and the lack of HD6 structural amphipathicity, while debilitating its productive interactions with many molecular, bacterial, and viral targets [41,42], is ideally suited for "cross-linking" HIV virions and the target cell. Further analysis of the molecular determinants mediating the HIV enhancing effect of HD5 and HD6 will provide a better understanding of the relationship between structure--and specific residues in particular--and the HIV enhancing function.

**Figure 7:**
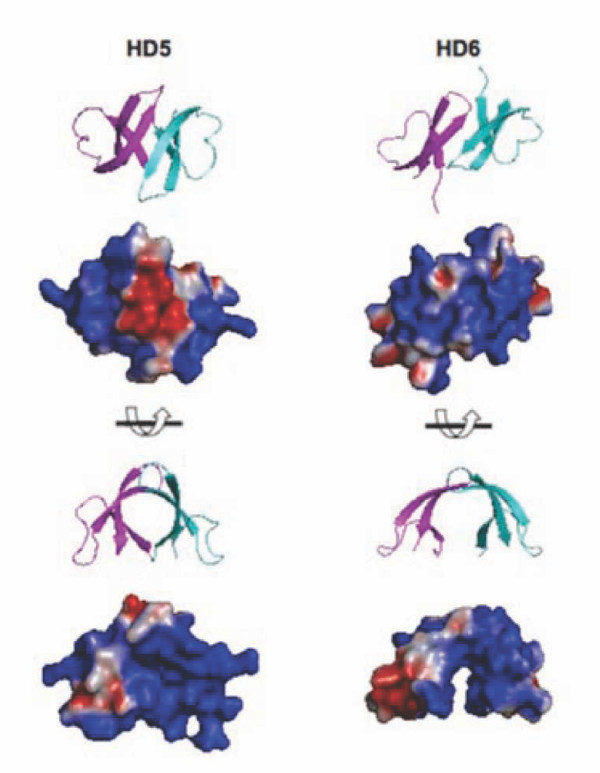
**Electrostatic surface potentials of HD5 and HD6 homodimers**. Monomers A and C of PDB 1ZMP were used to generate the HD5 homodimer, and monomers A and B of PDB 1ZMQ were used to generate the HD6 homodimer [35]. Electrostatic potentials were calculated using APBS [52] and displayed on the solvent-accessible surface. Electronegative and electropositive surfaces are colored red and blue, respectively, and contoured from -3 to +3 kT/e.

Heparin modulated the effect of HD5, but not HD6, on HIV infection. The net positive charge of HD5 (+4) is higher than that of HD6 (+2); thus, a simple net charge neutralization is unlikely to explain the inhibition of HD5-mediated HIV enhancement by heparin. We observed differences in their dimer structures and electrostatic surface potentials ([35] and Figure 7). The electrostatic surface potentials of HD5 and HD6 monomers were previously described [35]. We note that the HD5 and HD6 homodimers display significantly different electrostatic surface potentials from one another, and that HD6 dimerization generates an electropositive cleft not observed in the HD5 homodimer (Figure 7). Both charge and hydrophobicity are known to contribute to binding of a protein to heparin [43]. Hydrophobicity rather than cationicity has been recently shown to play a dominant role in the killing of Gram-positive bacteria, inhibition of anthrax lethal factor, and binding of HIV gp120 by HNP-1 [44]. While other defensins such as HNP-1, HNP-4, and HBD3 interact with heparin and heparan sulfate [45], the binding of HD5 and HD6 to heparin remains to be determined. Further studies on specific residues in defensins are required to elucidate the role of cationicity and hydrophobicity in the binding of defensins to heparin. In addition, our results suggest that heparin and HD5 may bind to the same regions of HIV gp120. Heparin is known to bind to the V3 loop and to the CD4 induced site of HIV gp120 [27,31,33,46]. Thus, identification of specific regions of HIV gp120 proteins that interact with HD5 and HD6 would likely clarify the interplay between defensins and polyanionic polymers such as heparin and polyanionic microbicides.

The semen-derived enhancer of viral infection peptide (SEVI) has been shown to significantly enhance HIV infectivity, implicating its involvement in sexual transmission of HIV at the mucosa [47]. SEVI promotes binding of HIV-1 R5 and X4 virus to target cells [47]. Polyanionic polymers including heparin and dextran sulfate, but not chondroitin sulfate, block the HIV enhancing effect of SEVI peptides [48]. We have previously shown that the HIV enhancing effect of HD5 and HD6 is more pronounced with R5 virus compared to X4 virus [17], suggesting the clinical significance of defensins as R5 viruses are almost exclusively detected upon sexual transmission. In contrast to SEVI peptides, HD5 and HD6 promoted HIV infectivity in the presence of these polyanionic polymers (albeit high concentrations of heparin inhibit HD5). After the disappointing results of trials using candidate polyanion microbicides, anti-retroviral drug based microbicides have become the current focus in microbicide development. A recent report indicated that a gel containing 1% tenofovir reduced HIV acquisition by an estimated 39% overall, and by 54% in women with high gel adherence [49]. Our studies on the interplay between defensins and HIV inhibitors, such as TAK779 and T20, suggest that the presence of sufficient amounts of HIV inhibitors during viral infection and high adherence are required to maintain the efficacy of topical microbicides in overcoming the HIV enhancing effect of endogenous peptides at the vaginal mucosa.

In conclusion, we demonstrated that HD5 and HD6 promoted HIV infectivity by enhancing the attachment of HIV to target cells. Understanding the complex functions of these mucosal host factors in HIV transmission is crucial for the development of new strategies for HIV prevention, especially in the setting of STIs.

## Materials and methods

### Reagents

HD5 and HD6, as well as linear unstructured forms of HD5 and HD6, [Abu]HD5 and [Abu]HD6, in which the six cysteine residues were replaced by isosteric α-aminobutyric acid (Abu), were chemically synthesized and folded as described previously [24]. The molecular mass of the peptides was verified by electrospray ionization mass spectrometry (ESI-MS) as described previously [24]. Both synthetic HD5 and HD6 were correctly folded as indicated by structural analysis by X-ray crystallography [35]. Heparin, chondroitin sulfate, dextran sulfate, and heparinase I were purchased from Sigma (St. Louis, IN).

### Cell culture

Peripheral blood mononuclear cells (PBMC) from normal healthy blood donors were isolated by Ficoll-Hypaque gradient centrifugation. CD4^+ ^T cells were isolated from PBMCs by negative selection using a CD4^+ ^T cell isolation kit from Miltenyi Biotech (Auburn, CA). The purity of cells was 98% based on flow cytometric analysis. CD4^+ ^T cells were stimulated with phytohemagglutinin (PHA) at 5 μg/ml and maintained in RPMI medium supplemented with 10% fetal bovine serum (FBS) and 25 units/ml IL-2 for 3 days at 37°C prior to viral infection. HeLa-CD4-CCR5 cells were provided by David Kabat (University of Oregon, Portland) and maintained in Dulbecco's minimal essential medium (DMEM) containing 10% FBS.

### HIV-1 infection

Replication-defective HIV-1 luciferase-expressing reporter viruses, pseudotyped with HIV-1_JR-FL _(gift of D. Littman, New York University) for a single-cycle infection assay, were produced as described previously [50,51]. Briefly, HEK293T cells were co-transfected with a plasmid encoding the envelope-deficient HIV-1 NL4-3 virus with the luciferase reporter gene inserted into *nef *(pNL4-3.Luc.R-E-, AIDS Research & Reference Reagent Program, ARRRP, National Institute of Allergy and Infectious Disease, National Institutes of Health, from N. Landau, New York University) and a pSV plasmid expressing the JR-FL glycoprotein. The supernatant was collected 48 hours after transfection, and filtered. Virus stocks were analyzed for HIV-1 p24 antigen by ELISA (SAIC Frederick, Frederick, MD). To produce HIV-1_JR-FL _pseudotyped viruses in the absence of serum, transfection was performed as described above. Transfected cells were incubated for 24 h, washed with PBS, and cultured in medium without serum for an additional 24 h prior to collecting viruses.

To assess whether defensins enhanced HIV infection by acting on the virions, serum-free pseudotyped HIV-1_JR-FL _luciferase reporter viruses were incubated with defensins at 20 μg/ml at 37°C for 1 hour. FBS at a final concentration of 10% (v/v) was added the defensin-virus mixture before addition to HeLa-CD4-CCR5 cells, seeded at 5 × 10^4 ^in a 48-well plate and grown for overnight. After 2 h incubation, cells were washed extensively and cultured for 48 hours before measuring of luciferase activity using Luciferase Substrate Buffer (Promega Inc). Luciferase activity (relative light units; R.L.U.) reflecting viral infection was measured on an EG & G (Berthold) MiniLumat LB9506 luminometer.

To determine the effect of defensins on the target cell, PHA-activated primary CD4+ T cells (1 × 10^6^) or HeLa-CD4-CCR5 cells (5 × 10^4^) were treated with defensins in the presence of FBS for 1 hour at 37°C, washed, exposed to pseudotyped HIV-1_JR-FL _luciferase reporter viruses for 2 hours, washed, and cultured for additional 48 hours.

To determine the effect of defensins on anti-HIV activity of HIV inhibitors, HeLa-CD4-CCR5 cells were pre-treated with or without TAK-779 (2 μM) or T-20 (200 nM) for 1 hour. Cells without HIV inhibitor treatment were included as a control. Serum-free pseudotyped HIV-1_JR-FL _virus (~10 ng p24 per sample) was incubated with HD5 or HD6 at 20 μg/ml at 37°C for 1 hour. The virus mixture was then added to cells in the presence or absence of inhibitors for 2 hours. After washing off unbound virus, infected cells were cultured in the absence (wash off) or presence (add back) of the inhibitors for 48 hours before measurement of luciferase activity.

To determine the effect of defensins on HIV infection in the presence or absence of soluble GAGs, serum-free HIV-1_JR-FL _pseudotyped luciferase reporter virus was incubated with or without HD5 or HD6 in the presence of soluble GAGs at varying concentrations at 37°C for 1 hour followed by HIV infection. The removal of cell-associated GAGs was performed by incubating with heparinase I (20 U/ml) for 2 hours at 37°C. Cells were washed with PBS three times before HIV infection.

### HIV attachment assay

HeLa-CD4-CCR5 cells were seeded at 5 × 10^4 ^per well in 48-well plates and cultured overnight. PHA-activated primary CD4+ T cells (5 × 10^5 ^per sample) were prepared as described above. Serum-free pseudotyped HIV-1_JR-FL _was pre-incubated in the absence or presence of defensins for 1 h at 37°C. FBS was added the virus mixture to a final concentration to 10% (v/v) before addition to cells. Cells were then incubated with virus for 2 hours at 4°C or 37°C. Cells were washed four times and lysed with 1% Triton X-100. Cell-associated HIV p24 antigen was measured by p24 ELISA (NCI, Frederick).

To access the effect of defensins on HIV attachment by FACS analysis, pseudotyped HIV-1_JR-FL _virus containing Vpr-GFP (25 ng p24) was incubated with or without defensins for 1 hour before exposure to EDTA-suspended HeLa-CD4-CCR5 cells (5 × 10^5 ^cell per sample) at 4°C for 2 hours. After washing off unbound virus, cells were fixed with 2% paraformaldehyde and analyzed on a FACScan (Becton Dickinson, CA). Results were analyzed with FlowJo Software (Tree Star, OR). To analyze the effect of defensins using microscopy, HeLa-CD4-CCR5 cells at 2.5 × 10^5 ^cells per well were seeded into a 4-well chamber slide and cultured overnight. The defensin-GFP virus mixture was added to the cells and incubated at 4°C for 2 hours. After washing off unbound virus, cells were fixed and mounted with VECTASHIELD HardSet mounting media with DAPI (Vector, CA) and visualized using Axioplan 2 (Zeiss, Germany). The images were analyzed using Volocity 5.2.1 (Perkin Elmer, MA).

## Competing interests

The authors declare that they have no competing interests.

## Authors' contributions

AR performed the experiments on HIV infection and HIV attachment by HIV p24 ELISA. JD performed the experiments on HIV infection and HIV attachment by FACS and microscopy, and prepared the manuscript; BB assisted in the preparation of recombinant viruses and HIV infection; YL performed statistical analysis; MN analyzed the surface charges of dimerized defensins and prepared the manuscript; WL prepared peptides, discussed the results, and was involved in manuscript preparation; TLC oversaw the entire project, designed experiments and prepared the manuscript. All authors read and approved the final manuscript.
